# Linear Frequency Modulation of NbO_2_-Based Nanoscale Oscillator With Li-Based Electrochemical Random Access Memory for Compact Coupled Oscillatory Neural Network

**DOI:** 10.3389/fnins.2022.939687

**Published:** 2022-06-30

**Authors:** Donguk Lee, Myonghoon Kwak, Jongwon Lee, Jiyong Woo, Hyunsang Hwang

**Affiliations:** ^1^Department of Materials Science and Engineering, Center of Single Atom-based Semiconductor Device, Pohang University of Science and Technology, Pohang, South Korea; ^2^School of Electronics Engineering, Kyungpook National University, Dague, South Korea

**Keywords:** oscillatory neural network, coupled oscillator, insulator-metal transition, electro-chemical random access memory, spoken vowel, classification of spoken vowel

## Abstract

Oscillatory neural network (ONN)-based classification of clustered data relies on frequency synchronization to injected signals representing input data, showing a more efficient structure than a conventional deep neural network. A frequency tunable oscillator is a core component of the network, requiring energy-efficient, and area-scalable characteristics for large-scale hardware implementation. From a hardware viewpoint, insulator-metal transition (IMT) device-based oscillators are attractive owing to their simple structure and low power consumption. Furthermore, by introducing non-volatile analog memory, non-volatile frequency programmability can be obtained. However, the required device characteristics of the oscillator for high performance of coupled oscillator have not been identified. In this article, we investigated the effect of device parameters of IMT oscillator with non-volatile analog memory on coupled oscillators network for classification of clustered data. We confirmed that linear conductance response with identical pulses is crucial to accurate training. In addition, considering dispersed clustered inputs, a wide synchronization window achieved by controlling the hold voltage of the IMT shows resilient classification. As an oscillator that satisfies the requirements, we evaluated the NbO_2_-based IMT oscillator with non-volatile Li-based electrochemical random access memory (Li-ECRAM). Finally, we demonstrated a coupled oscillator network for classifying spoken vowels, achieving an accuracy of 85%, higher than that of a ring oscillator-based system. Our results show that an NbO_2_-based oscillator with Li-ECRAM has the potential for an area-scalable and energy-efficient network with high performance.

## Introduction

Von-Neumann computing architecture has a drawback of inefficient data transportation between memory and processor, referred to Von-Neumann bottleneck. Thus, bioinspired new computing architecture has gained enormous attention, anticipating low power consumption, and parallel processing (Mead, 1990). Several artificial neural networks (ANNs) have been developed inspired by brain function. Among these, an oscillatory neural network (ONN) is composed of coupled oscillators, motivated by the synchronization of oscillatory neural signals to cognize complex information in neural binding. Oscillator-based network shows complex non-linear dynamics, which can be utilized in various applications such as classification of clustered data (Romera et al., 2018; Dutta et al., 2019), associative memory system (Levitan et al., 2012; Shibata et al., 2012), pattern recognition (Nikonov et al., 2015; Vodenicarevic et al., 2016), and Nondeterministic polynomial (NP)-hard problem solver (Parihar et al., 2017; Dutta et al., 2021).

For the classification of clustered data, a coupled oscillator-based spoken vowel classification system has been reported (Romera et al., 2018; Dutta et al., 2019). Spoken vowel signals have formant frequencies, which are peaks in the frequency spectrum. The set of formant frequencies depends on the vowel, which is a feature of the input signal. Thus, input vowel signals are transformed into a two-dimensional domain in the form of frequency, spreading multiple frequency clusters. The frequencies are injected into coupled oscillators network, resulting in a synchronization map with trained nature frequencies of the network. Consequently, vowels are clustered according to states in the synchronization map. Compared to conventional ANN such as multilayer perceptron, recurrent neural network (RNN), and long short-term memory (LSTM), coupled oscillator network has a simple structure and a small number of trained parameters with a comparable recognition rate (Romera et al., 2018). Therefore, an oscillator-based spoken vowel classification system has the advantage of computing power efficiency.

To implement these systems by the conventional CMOS technology, an oscillator is composed of a ring oscillator and a current-based digital-to-analog converter (DAC) to tune oscillation frequency to the trained value (Nikonov et al., 2020). Thus, many transistors and high operation power are required, resulting in the limitation of a large-scale system.

In this regard, spin-torque oscillator (STO) (Romera et al., 2018), insulator-metal transition (IMT) device (Lee et al., 2018; Dutta et al., 2019), and ovonic threshold switch (OTS) device (Lee et al., 2020) have been reported to overcome the limitations of conventional CMOS-based oscillator. Among the abovementioned oscillators, the IMT-based oscillator has a simpler structure and lower power consumption than the others. In particular, the NbO_2_-based IMT oscillator shows stable oscillation due to the drift-free threshold switching characteristics of NbO_2_ (Park et al., 2017). In an IMT oscillator, the oscillation frequency is determined by the resistance of the load resistor (Chen et al., 2016). Therefore, frequency controllability can be obtained by adjusting variable resistors such as transistor and resistive random access memory (RRAM) (Lee et al., 2018; Dutta et al., 2019). An additional memory device is required to store trained natural frequencies of coupled oscillators. NbO_2_-based IMT oscillator with non-volatile RRAM and Li-based electro-chemical random access memory (Li-ECRAM) has frequency storable characteristics (Lee et al., 2018; Lee et al., 2022). Thus, compared to the volatile transistor as load, non-volatile memory is advantageous in terms of the simplicity of hardware. Although frequency storable IMT oscillator with non-volatile analog memory has been proposed, the effects of device parameters of IMT and memory device are not identified in terms of network performance.

This study investigated the effect of device parameters on the performance of coupled oscillator networks to classify clustered data. As a result, the Li-ECRAM device is an appropriate oscillation load for high learning accuracy. Finally, we evaluated spoken vowel classification based on a nanoscale oscillator with NbO_2_ and Li-ECRAM, showing high learning accuracy.

## Materials and Methods

### Measurement and Simulation Platform

The electrical characteristics of NbO_2_-based IMT device and Li-ECRAM were measured using a Keysight B1500A semiconductor device parameter analyzer with Waveform Generator/Fast Measurement Unit (WGFMU) module. Input current pulses for programming conductance of Li-ECRAM were generated by Keithley B2635B. We used a Keysight 81160A Pulse Function Arbitrary Noise Generator to generate injected sine waves. Output waveforms of oscillators were measured by a Keysight DSOX4154A oscilloscope. Simulation Program with Integrated Circuit Emphasis (SPICE) simulations of coupled oscillators were performed by using Synopsis HSPICE 2020.06.

### NbO_2_-Based Insulator-Metal Transition Device

**FIGURE 1:**
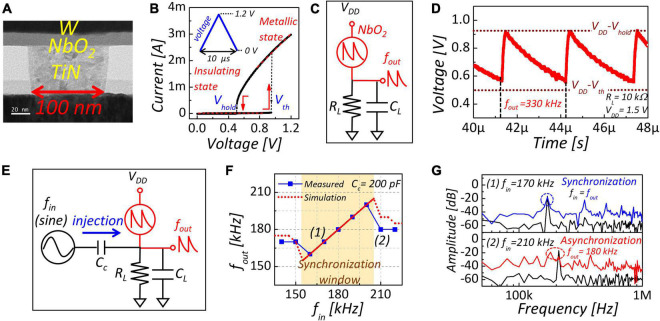
**(A)** TEM image of NbO_2_-based IMT device. **(B)** I–V characteristic of the NbO_2_-based IMT device. **(C)** Schematic diagram of NbO_2_-based oscillator. **(D)** Output waveform of NbO_2_-based oscillator when load resistor is 10 kΩ. **(E)** Schematic diagram of injection locking with input signal. **(F)** Output frequency as a function of input frequency. Colored region is synchronization window. **(G)** Fast Fourier transformation (FFT) result of input and output frequency. In FFT analysis, sampling rate is 400 Ms^−1^.

The NbO_2_-based IMT device with a metal-oxide-metal (MIM) stack has been fabricated using the following process. First, a 20-nm-thick NbO_2_ layer was fabricated on a TiN plug with a 100 nm diameter, as shown in Figure 1A. For a thin NbO_2_ deposition, we used radio frequency (RF) magnetron reactive sputtering with Nb metal target in an O_2_/Ar gas mixture in the ratio of 1/15 at room temperature. Then, a W top electrode was deposited by direct current (DC) magnetron sputtering. Figure 1B shows the hysteresis current–voltage (I–V) characteristics of the NbO_2_ device using a triangle shape with a 10 μs width. The IMT device transforms from an insulating state to a metallic state when the applied voltage exceeds the threshold voltage (V_th_). In contrast, after transition, if the applied voltage is smaller than the hold voltage (V_hold_), the IMT device goes back to the initial insulating state.

### NbO_2_-Based Insulator-Metal Transition Oscillator

Figure 1C presents a schematic of an NbO_2_-based IMT oscillator. When the load resistor is connected, two stable states exist owing to the hysteresis characteristics of NbO_2_ in the following condition (Chen et al., 2016):


(1) $$\frac{R_{i}}{R_{i}+R_{L}}>\frac{V_{\mathrm{th}}}{V_{\mathrm{DD}}}\,a\,n\,d\,\frac{R_{m}}{R_{m}+R_{L}}<\frac{V_{\mathrm{hold}}}{V_{\mathrm{DD}}}$$

where *R*_*i*_ is the insulating resistance of NbO_2_, *R*_*m*_ is the metallic resistance of NbO_2_, *R*_*L*_ is load resistance, and *V*_*DD*_ is supply voltage.

Therefore, the oscillator’s output showed self-sustained oscillation between V_DD_-V_th_ and V_DD_-V_hold_, accompanying the charging and discharging of the load capacitor (*C*_*L*_), as shown in Figure 1D. The oscillation frequency is determined by the charging and discharging time. When the resistance condition is *R_*i*_* > > *R_*L*_* > > *R*_*m*_, the charging time is significantly shorter than the discharging time. Thus, the oscillation frequency is dominant on discharging time and represented as follows:


(2) $$f=\frac{G_{L}}{C_{L}\,\ln\left(\frac{V_{\mathrm{DD}}-V_{\mathrm{hold}}}{V_{\mathrm{DD}}-V_{\mathrm{th}}}\right)}$$

where G_L_ is the conductance of the load resistor (= 1/R_L_).

We then evaluated injection locking to external sine wave, a crucial phenomenon of coupled oscillator-based systems, as shown in Figure 1E. Injection locking is a phenomenon in which the frequency of an oscillator is synchronized (locked) with injected frequency when the injection frequency is within the synchronization range (locking range). Sinewave with 1.4 V high level and 1.1 V low level was injected through a coupled capacitor (*Cc*). When frequency within the synchronization window (*W*) close to the natural frequency (170 kHz) was injected, the output frequency is locked to the input frequency, as shown in the colored region of Figure 1F. Thus, input and output frequency was synchronized, resulting in equal peak frequency in FFT results of input and output oscillation, as shown in Figure 1G. In contrast, an input frequency greater than 210 kHz, out of the synchronization window, caused a mismatch with the output, meaning asynchronization.

### Coupled Oscillator-Based Classification of Clustered Data

**FIGURE 2:**
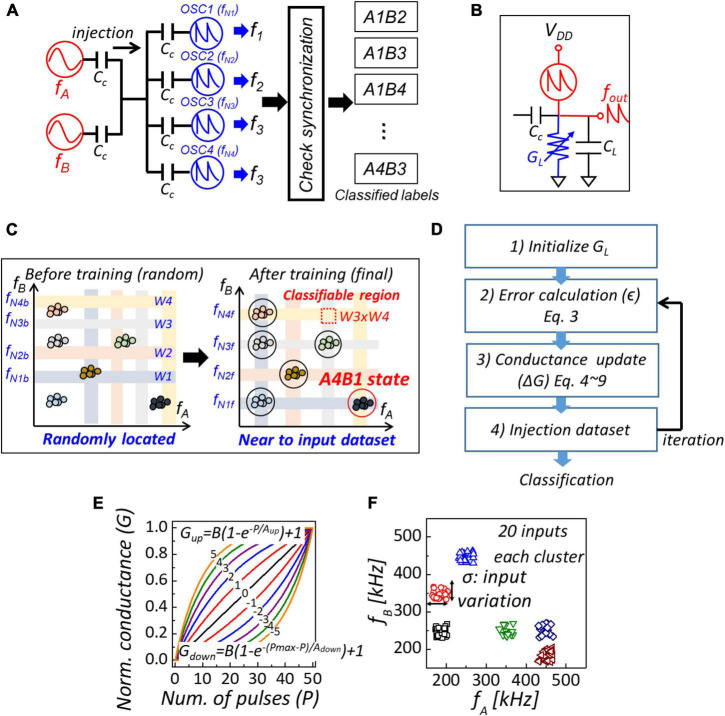
**(A)** Four-coupled oscillators configuration of a clustered data classification using **(B)** NbO_2_-based oscillator with analog memory device. **(C)** Synchronization map of before and after training. W is synchronization window and classifiable region is overlapped area with synchronization windows (W × W). **(D)** Flow chart of conductance update for classification of clustered data. **(E)** Normalized conductance response to applied number of pulses. **(F)** Training and test dataset with input variation.

Based on the injection locking phenomenon of coupled oscillators when input within the synchronization window is injected, we investigated the effect of device parameters on 4-coupled oscillators with NbO_2_ devices for classification of clustered data by SPICE simulation, as shown in Figure 2A. In the simulation, we used Verilog-A NbO_2_ compact model fitted from I to V characteristics (Lee et al., 2019), as shown in Figure 1B, load capacitance (C_L_) is 400 pF, and coupling capacitance (C_c_) is 200 pF. Furthermore, we introduced analog memory as a load resistor to obtain frequency tunability as shown in Figure 2B. Two-dimensional clustered input frequencies (f_A_, f_B_) are injected simultaneously through coupling capacitors. After injection, output frequencies (f_1_–f_4_) of each oscillator are checked synchronizations with the input frequencies through synchronization-detecting circuits (Vodenicarevic et al., 2016). Then, input frequencies are mapped and labeled according to the synchronization state. In this system, classifiable regions correspond to overlapped areas with synchronization windows in the synchronization map. For example, the A4B1 region representing oscillators 4 and 1 is synchronized with input frequencies f_A_ and f_B_, respectively, as shown in Figure 2C. Input data within the region are classified as the same cluster. Therefore, for accurate classification, oscillation frequencies must be trained for classified regions to cover input data. We trained the natural frequencies (f_N_) of oscillators by gradient descent algorithm (Romera et al., 2018; Dutta et al., 2019). The conductance of analog memory composing NbO_2_-based oscillators must be trained to obtain optimized nature frequencies. Figure 2D shows the flowchart for the training of conductance. First, the conductance of load resistors was randomized within a limited range (40 μS–200 μS). Then, training inputs are injected to calculate the error (ϵ) between input frequencies and corresponding output frequencies (f_out_). Training input is the input frequency set [f_A_, f_B_] and label of oscillators (*L*) with which oscillator the input is synchronized. For example, labels for input corresponding to A1B4 region of synchronization map are L_A_ = [1, 0, 0, 0] and L_B_ = [0, 0, 0, 1]. Therefore, the error is calculated as follows:


(3) $$\epsilon_{A}={\left[L_{A}\right]}^{T}\,\left[f_{\mathrm{out}}-f_{A}\right],\epsilon_{B}={\left[L_{B}\right]}^{T}\,\left[f_{\mathrm{out}}-f_{B}\right]$$

Mean-squared error was used as a loss function (*L*):


(4) Ltotal=∑ϵtotal2/N=∑(ϵA+ϵB)2/N

where N is the number of oscillators (= 4). According to equation (2), the natural frequency of coupled oscillators (*f*_*N*_) is linearly proportional to the conductance of the load resistor (*G*_*L*_). Then, the amount of conductance update was calculated by gradient descent as follows:


(5) $$\Delta \,G_{i}=\eta \,\delta \,L_{\mathrm{total}}/\delta \,G_{i}$$

where η is the learning rate and *G*_*i*_ is the load conductance of i^th^ oscillator. In a perceptron neural network with analog memory-based synapse devices, such as RRAM and phase change random access memory, the synaptic weight corresponding to the conductance of analog memory is updated by applying identical pulses proportional to the amount of weight update. An identical pulse scheme is a practical method, because a non-identical pulse scheme requires heavier circuitry than the identical pulse scheme (Tsai et al., 2018). Therefore, the applied number of pulses to update the conductance of analog memories is calculated as follows:


(6) $$P_{i}=\mathrm{round}\,\left(\Delta \,G_{i}\right)$$

where *P*_*i*_ is the applied number of pulses of i*^th^* analog device. The conductance update process is iterated until the conductance of analog memories saturates to the optimal value. The conductance change characteristics of analog memory under identical pulses are essential to update accuracy, and conductance modulation behavior was modeled as a normalized exponential function (Chen et al., 2017).


(7) $$G_{\mathrm{up}}=B\,\left(1-e^{-\frac{P}{A_{\mathrm{up}}}}\right)+1$$

(8) $$G_{\mathrm{down}}=B\,\left(1-e^{\frac{P-P_{\max}}{A_{\mathrm{down}}}}\right)+1$$

(9) $$B=1/\left(1-e^{\frac{-P_{\max}}{A}}\right)$$

where G_*up*_ is a function of increasing conductance, G_down_ is a function of decreasing conductance, A is the parameter determining non-linear behavior, and B is different in G_up_ and G_down_, as shown in Figure 2E. Then, we considered fitting function in simulation. Figure 2F shows the training and test datasets, with 20 inputs for each cluster (a total of 5 clusters) with variation (σ).

### Effect of Non-linearity (A)

**FIGURE 3:**
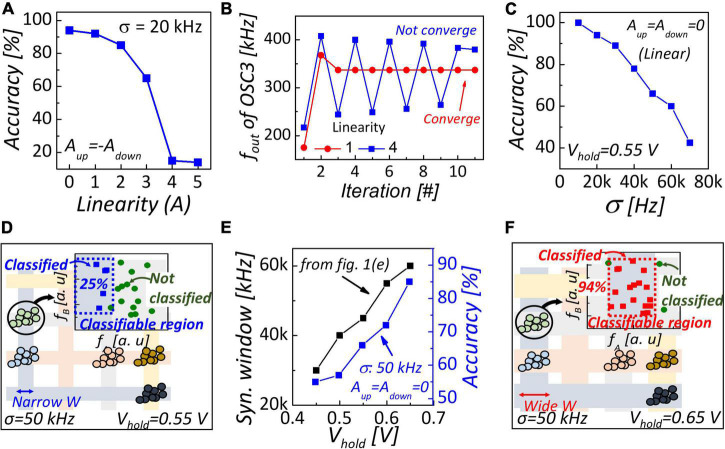
**(A)** Classification accuracy as a function of non-linearity. The non-linearity of the up and down conductance response is symmetric. **(B)** Output frequency of 3rd oscillator as a function of training iteration in various non-linearity. **(C)** Classification accuracy as a function of input variation. To investigate only the effect of input variation, conductance response is linear with the number of pulses. **(D)** Synchronization map with narrow synchronization window (V_*hold*_ = 0.55 V); 25% of clustered data was within the classifiable region due to narrow W. **(E)** Synchronization window and classification accuracy with various hold voltage of NbO_2_ device. Synchronization window was simulated in the configuration shown in Figure 1E. **(F)** Synchronization map with wide synchronization window (V_*hold*_ = 0.65 V). 94% of clustered data can be classified owing to wide classifiable region.

To investigate the effect of non-linearity on network performance, we evaluated classification accuracy with various non-linearities, as shown in Figure 3A. We assumed that the non-linearity of up and down conductance response was symmetric owing to confirm non-linearity only. Classification accuracy was degraded as the non-linearity was higher. If the non-linearity is high, conductance changes significantly even if the number of pulses is small. Consequently, the output frequencies proportional to the conductance of analog memory do not converge to the optimal value when non-linearity is high, as shown in Figure 3B. Therefore, linear conductance modulation under identical pulses is crucial to improve classification accuracy.

### Effect of Input Variation and Hold Voltage

Figure 3C shows the classification accuracy as a function of input variation. High input variation worsened accuracy because the classifiable region cannot cover clustered data. When input variation was 50 kHz, only 25% of clustered data were covered by the classification region as shown in Figure 3D. To widen the classifiable region, a wide synchronization window is required. According to Alder’s equation (Bhansali and Roychowdhury, 2009) representing an injection locking phenomenon, locking range *f*_*L*_ corresponding to synchronization window is inversely proportional to the amplitude of the oscillator:


(10) $$f_{L}\propto V_{i}/V_{\mathrm{osc}}$$

where *V*_*i*_ is the amplitude of injected sinuous signal and *V*_*osc*_ is the amplitude of the oscillator. As shown in Figure 1D, the amplitude of the NbO_2_-based oscillator is V_th_-V_hold_. Thus, the synchronization window widened with and enlarged V_hold_, improving classification accuracy, as shown in Figure 3D. Enlarged V_hold_ (= 0.65 V) leads to a broad classifiable region, which covers 94% of clustered data, as shown in Figure 3F. Therefore, immunity of input variation can be obtained by an enlarged V_hold_ of NbO_2_.

### Li-Based Electrochemical Random Access Memory

**FIGURE 4:**
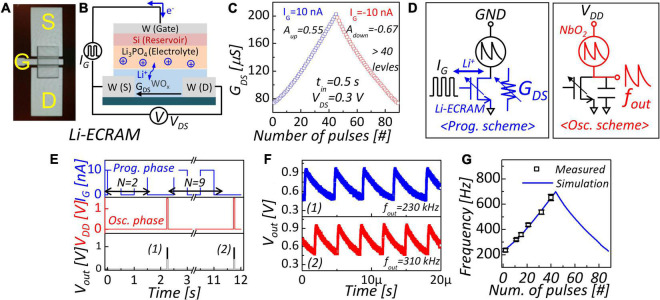
**(A)** Optical microscope image and **(B)** device structure of Li-ECRAM. **(C)** Conductance response to identical pulses. Low non-linearity (A_*up*_ = 0.55, A_*down*_ = −0.67) is obtained. **(D)** Frequency programming scheme and oscillator scheme of NbO_2_-based oscillator with Li-ECRAM. **(E)** Real-time measurement of oscillation frequency modulation. N represents applied number of pulses in programming scheme. **(F)** Magnified figure of output waveform in **(E)**. **(G)** Frequency modulation with applied number of pulses.

In terms of conductance linearity, Li-ECRAM is the most appropriate analog memory as load. Since Li-ECRAM exhibits linear conductance modulation (Fuller et al., 2017; Tang et al., 2018), it shows linear frequency modulation in an NbO_2_-based oscillator with the device (Lee et al., 2022). A three-terminal Li-ECRAM device was fabricated on a SiO_2_ wafer. First, W source and drain were deposited by DC magnetron sputtering. The distance between source and drain corresponding to channel length was 100 nm. Then, a 50-nm-thick WO_3_ channel material was deposited by RF magnetron reactive sputtering with W metal target in an O_2_/Ar gas mixture in the ratio of 1/5 at room temperature. Consecutively, 100-nm-thick Li_3_PO_4_ electrolyte and 30-nm-thick Si reservoir were deposited by RF magnetron sputtering. Finally, the W gate was deposited by DC magnetron sputtering. An optical microscope image of the fabricated device is shown in Figure 4A. Figure 4B shows the device structure of Li-ECRAM and the bias schematic for channel conductance modulation. When a positive current is applied, Li-ions in electrolyte were injected into the channel. Then, W^6+^ valance state changes to W^5^ +, increasing channel conductance (Niklasson et al., 2004). In contrast, when a negative current is applied, Li-ions in the channel are extracted from the electrolyte, decreasing channel conductance. Figure 4C shows the conductance response to applied identical gate current pulses. The amplitude of input gate pulses (I_G_) for changing conductance up and down was 10 and –10 nA, respectively, and the pulse width was 0.5 s. Drain–source voltage (V_DS_) was applied to measure channel conductance (G_DS_). Conductance change has a reasonably linear relationship with the applied number of pulses, showing a low non-linearity factor (A_up_ = 0.55, A_down_ = −0.67). The conductance switching of LI-ECRAM is driven by the applied charge. Conductance response is linearly proportional to the number of pulses related to the applied ones (Fuller et al., 2017; Tang et al., 2018). As shown in Figure 3A, accuracy degradation is slightly degraded in using Li-ECRAM as oscillation load compared to ideal linear case.

### NbO_2_-Based Oscillator With Li-Based Electrochemical Random Access Memory Load

In the configuration of the NbO_2_-based oscillator with Li-ECRAM, two devices are connected in series. Figure 4D shows the operation scheme for frequency programming and oscillation. In the programming scheme, gate pulses for modulation of channel conductance are applied to the gate side of Li-ECRAM. The current is applied to the gate to determine conductance changes, and supply voltage is not applied (GND). In the oscillation scheme, the supply voltage is applied, and oscillation occurs, as shown in Figure 1D. Figure 4E shows the real-time measurement of frequency programming (programming-oscillation-programming-oscillation). First, we applied two programming pulses and then confirmed 230 kHz oscillation, as shown in Figure 4F. Nine programming pulses were applied, and the oscillator exhibited 310 kHz oscillation. In this way, the oscillation frequency can be tuned. As a result, the oscillation frequency is a function of the applied number of pulses, as shown in Figure 4G.

## Results

### Spoken Vowel Classification Using NbO_2_-Based Oscillator With Li-Based Electrochemical Random Access Memory

**FIGURE 5:**
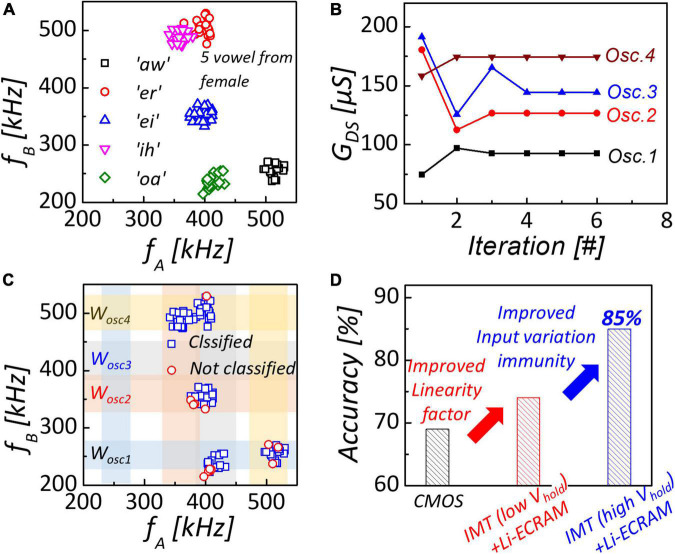
**(A)** Dataset of spoken vowel. Formant frequencies of spoken vowel is processed by linear transformation to match frequency of NbO_2_-based oscillator with Li-ECRAM. **(B)** Conductance of Li-ECRAMs as function of training iteration. After five iterations, channel conductance of Li-ECRAMs converge to the optimal value. **(C)** Synchronization map after training. Most of clustered data are covered by classifiable region. **(D)** Introducing Li-ECRAM and adjusting Vhold of NbO_2_, 85% classification accuracy can be obtained.

Using four-coupled oscillators with NbO_2_-based oscillators connected to Li-ECRAM, we evaluated the classification of the spoken vowel. American English vowel dataset with 5 vowels from 20 different females was used in the simulation (Hillenbrand, 1995). To utilize a coupled oscillator network for the classification of clustered data, input frequencies must be within the frequency range of an NbO_2_-based oscillator with Li-ECRAM. Thus, linear transformation must process formant frequencies of spoken vowels to match with the oscillator’s frequency range (Romera et al., 2018; Dutta et al., 2019), as shown in Figure 5A. The conductance of Li-ECRAMs was trained by the gradient descent learning rule mentioned in the “NbO_2_-based IMT oscillator” section and converged after five cycles, as shown in Figure 5B. After training, oscillation frequencies were programmed for the classifiable region of the synchronization map to catch input vowel data, as shown in Figure 5C. Figure 5D shows the classification accuracy of networks with various oscillators. In this comparison, the CMOS-based ring oscillator exhibited a non-linearity of 1.78, extracted from frequency modulation characteristics with digital input code to DAC (Nikonov et al., 2020). Due to the high non-linearity of the CMOS-based oscillator, classification accuracy is low (69%). Introducing Li-ECRAM as oscillation load with low non-linearity (0.55/–0.67), the accuracy improved by 74%. Furthermore, an accuracy of 85% was obtained by adjusting the V_*hold*_ of NbO_2_ for input variation immunity. In a multilayer perceptron with a similar number of trained parameters to an oscillator-based network, only an accuracy below 65% was achieved (Romera et al., 2018).

### Benchmark

We benchmarked oscillators for the coupled oscillator-based spoken vowel classification system as shown in Table 1. The small number of components means the scalability of the oscillator. CMOS-based ring oscillator has additional circuitry to tune the oscillation frequency (buffer, DAC) (Nikonov et al., 2020). STO consists of a magnetic tunnel junction (MTJ) and a bias tee to separate the injection input current (Romera et al., 2018). However, IMT oscillators [VO_2_-based (Dutta et al., 2019) and our proposed oscillator] require only a TS device, an oscillation load (NMOS, Li-ECRAM), and a capacitor. Linear frequency modulation corresponds to the linear conductance change of our proposed oscillator. CMOS-based oscillator shows non-linear frequency modulation with digital cord input to a DAC. The VO_2_-based oscillator is also not linearly proportional to the gate voltage because the channel resistance is not linearly related to the gate voltage. STO shows slight linear frequency modulation to the input current amplitude. However, the proposed oscillator has linear frequency modulation characteristics. The frequency programmability is the ability to store frequency itself. Frequencies of each oscillator must have trained values in the classification process. In cases of CMOS-based, STO, and VO_2_-based oscillators, information about the amplitude of analog input for frequency tuning is stored in additional memory devices. However, in the case of our oscillator, the conductance of Li-ECRAM related to frequency can be stored itself. Therefore, our proposed oscillator has a simpler structure than other reported oscillators owing to the simple configuration and unnecessariness of DAC and additional memory devices. Finally, our most uncomplicated hardware system has comparable classification accuracy to other systems. Therefore, our classification system with a simple configuration, area, and energy-efficient oscillator promises for large-scale hardware implementation.

**TABLE 1:** Benchmark of oscillators for spoken vowel classification.

	CMOS-based ring oscillator (Nikonov et al., 2020)	Spin-torque oscillator (STO) (Romera et al., 2018)	VO_2_-based IMT oscillator (Dutta et al., 2019)	This work
Components	Ring oscillator., DAC	MTJ, Bias tee	NMOS, VO_2_, Capacitor	Li-ECRAM, NbO_2_, capacitor
Input for frequency tuning	Digital cord	Current	Gate voltage	Identical pulse
Use of DAC	O	O	O	X
Linear frequency modulation	X	O	X	O
Frequency programmability	X	X	X	O
Classification accuracy of spoken vowel	69%	89%	90.5%	85%

## Conclusion

In this study, we identified the effect of device parameters of IMT oscillator with non-volatile analog memory device as oscillation load on the performance of coupled oscillator network. Non-linear conductance response of analog memory to identical pulses causes a divergence of conductance update in training iteration. Thus, the linear conductance response of analog memory to programming pulses was essential for the accurate training of natural frequencies. Furthermore, a narrow classifiable region of the synchronization map proportional to the synchronization window cannot cover injected input with large variation, reducing classification accuracy. Large V_*hold*_ inducing a large oscillation amplitude is required to widen the classifiable region. As a result of the investigation, Li-ECRAM as oscillation load is the potential for high network performance owing to linear conductance modulation characteristics. Finally, we evaluated the oscillatory network for spoken vowel classification with an NbO_2_-based IMT device and Li-ECRAM, achieving high classification accuracy (85%).

## Data Availability Statement

The original contributions presented in this study are included in the article/supplementary material, further inquiries can be directed to the corresponding author/s.

## Author Contributions

All authors listed have made a substantial, direct, and intellectual contribution to the work and approved it for publication.

## Conflict of Interest

The authors declare that the research was conducted in the absence of any commercial or financial relationships that could be construed as a potential conflict of interest.

## Publisher’s Note

All claims expressed in this article are solely those of the authors and do not necessarily represent those of their affiliated organizations, or those of the publisher, the editors and the reviewers. Any product that may be evaluated in this article, or claim that may be made by its manufacturer, is not guaranteed or endorsed by the publisher.
